# Improved Quality of Corn Silage When Combining Cellulose-Decomposing Bacteria and* Lactobacillus buchneri* during Silage Fermentation

**DOI:** 10.1155/2019/4361358

**Published:** 2019-02-17

**Authors:** Fanfan Zhang, Xuzhe Wang, Weihua Lu, Feifei Li, Chunhui Ma

**Affiliations:** Department of Animal Science, College of Animal Science and Technology, Shihezi University, Shihezi 832000, Xinjiang Province, China

## Abstract

This study aimed to investigate the effects of the combined use of cellulose-decomposing bacteria (CDB) and heterolactic lactic acid bacteria (LAB) on corn silage fermentation. Fresh maize was treated with heterolactic LAB or CDB combined with heterolactic LAB inoculants or without any treatment. Chemical and microbiological analyses were conducted at specific times after ensiling. A comprehensive value evaluation was conducted using the principal component analysis model. Although all treatments significantly affected the microorganism numbers during fermentation, the numbers of aerobic bacteria, LAB, yeast, and molds in the groups with combined CDB and LAB were significantly higher than those in the group with LAB only (*P* < 0.05). All treatments regulated the silage CO_2_ production. Each treatment had different effects on the nutrient degradation rate. Based on a comprehensive evaluation, the CDB and heterolactic LAB combination had the best effect on the ensiling process in improving the quality and feed value of corn silage.

## 1. Introduction

Silage is one of the most important animal forage for ruminants, having the advantages of providing less nutrient loss, good palatability, and high digestibility [1]. The principle of ensiling involves the conversion of water-soluble carbohydrates (WSCs) into organic acids (mainly lactic acid) by lactic acid bacteria (LAB) under an anaerobic environment to rapidly reduce the silage pH. As a result, decomposition of the nutrients is inhibited and the storage time of the forage is extended through its preservation from spoilage microorganisms. However, the condition of the silage has a significant impact on its quality. Owing to the anaerobic environment, substrate content, and WSCs, the silage day matter loss is always over 20% [2]. In addition, the increased content of neutral detergent fibers (NDF) in corn silage may inhibit the absorption and utilization of other nutrients [3]. Therefore, in order to effectively accelerate the development of agriculture and animal husbandry, improvements of the quality and fermentation of silage are crucial.

At present, LAB are commonly applied as the main microbial inoculant for improving the nutritional quality of fermented silage [4]. LAB addition can increase the dry matter recovery rate, thereby improving the quality of the fermented silage [5]. The metabolic product, acetic acid, produced by heterolactic LAB, can effectively affect the metabolism of yeasts and promote the stability of the silage in an aerobic environment to slow its deterioration. However, the heterolactic LAB transform lactic acid inefficiently and consume too much energy in the process, resulting in a certain amount of nutrient loss [6].

The use of heterolactic LAB as a silage additive has become common practice in recent years. However, the effectiveness of mixed LAB in silage fermentation is associated with the varieties of LAB strains and feeds used, where the combination needs to be modified according to the actual conditions [7, 8].

The addition of both LAB and cellulase during ensiling can promote lactic acid fermentation and delay secondary fermentation, thereby improving the digestibility of the silage [9–12]. However, the enzyme preparation has not been widely used because of its high cost and cumbersome preparation [13]. On the other hand, cellulose-decomposing bacteria (CDB) can increase the efficiency of substrate utilization by LAB, thereby promoting fermentation. The addition of CDB to dairy cow diets was shown to significantly increase the dry matter digestibility [14] and the quality of fermented cassava feed [15]. Although the positive effect of CDB on the quality of fermented products has been proved, details about the changes of the CDB and their effects on the performance of the LAB during the ensiling process are still unclear.

In this study, corn silage was fermented using both CDB and heterolactic LAB as a multispecies inoculant to investigate the effect of combined inoculants on the nutritional quality, fermentation characteristics, and microbial content of whole-plant corn silage. In addition, analyses of the aerobic stability and semi* in vitro* digestion of different fermentation combinations were performed to explore the combined effects of bacterial strains on the silage quality.

## 2. Materials and Methods

### 2.1. Maize and Bacterial Strains

Maize was planted at the pasture experimental station of Shihezi University in Xinjiang (N44°20′ E88°30′ H420 m). The local area is a typical continental arid climate with an annual average temperature of 7.5-8.2°C and an annual precipitation of 180-270 mm. The annual evaporation amount is 1000-1500 mm, the annual sunshine time is 2721~2818 h, and the frost-free period is 147-191 d. The local soil is heavy loam soil. The pH of the cultivated soil layer is 6.44, the organic matter content is 15.5 g/kg, the alkali nitrogen content is 16.8 g/kg, and the available phosphorus content is 0.54 g/kg. The fore-growing crop is cotton (*Anemone vitifolia Buch*). The maize planting time is from April 10, 2015 to August 20, 2015 with a growing season of 112 d. Maize (*Zea mays* ‘Xingsiyu No. 10') was wide-narrow row (60 cm + 40 cm) planted and was harvested at the early dough stage, chopped into approximately 2-cm pieces with a laboratory-type chopper, and stored at room temperature (23–30°C) for ensiling.

The CDB* Bacillus subtilis *(Cat. No: ACCC 19374),* Aspergillus niger *(Cat. No: ACCC 30134), and* Trichoderma viride *(Cat. No: ACCC 30595) were purchased from China Agricultural Culture Collection of China (ACCC). The heterolactic LAB* Lactobacillus buchneri* (Cat. No: CICC 20293) was purchased from China Center of Industrial Culture Collection (CICC).

### 2.2. Silage Preparation

On the day of maize harvesting, the silage was prepared using the vacuum bag (40×50 cm) method. Three different treatments were applied to the fresh materials: (1) blank control without any bacterial agent (K treatment); (2) heterolactic LAB (Y treatment); and (3) CDB (X) + Y (YX treatment). For the Y treatment,* Lactobacillus buchneri* was added at the concentration of 4.7 × 10^5^ cfu g^–1^ wet forage. For the X treatment,* Aspergillus niger*,* Trichoderma viride*, and* Bacillus subtilis* were added at the ratio of 2:1:1, respectively, at 0.3% of the total additive amount.

Four vacuum bags per treatment for each sampling time were prepared for chemical and microbiological analysis on days 2, 4, 8, 10, 15, 25, 35, 45, and 60 after ensiling. At the end of the ensiling period of 60 days, the silages were subjected to an aerobic stability test.

### 2.3. Microbiological Analysis Procedures

The aerobic bacteria, LAB, yeast, and molds in the silage were enumerated using the traditional plate counting method. In brief, the bacterial solution was inoculated onto plates with various selection media for incubation at strain-specific temperatures: nutrient agar (30°C) for the aerobic bacteria, de Man–Rogosa–Sharpe agar (37°C) for the lactobacilli, malt extract agar (25°C) for the yeast, and soybean casein digest agar (25°C) for the molds. The plate with the number of colonies between 30 and 300 was taken as the effective counting plate. The colony forming unit (cfu) is calculated as the number of microorganisms (cfu g^–1^ wet forage) = number of colonies × dilution factor × 1000 uL / coated sample volume (uL).

### 2.4. Chemical Analysis Procedures

Fermented silage (50g) was taken from each silo at the indicated time points and followed by adding 100 g of distilled water and macerating at 4°C for 24h. Then, the extracts were filtered using two layers of cheesecloth and two pages of filter paper. The filtrate was stored at -20°C prior to chemical analyses.

The pH value of the fermented silage was measured using a pH meter (PHS-25, Shanghai Precision & Scientific Instrument Co., Ltd., Shanghai, China). Lactic acid was determined using hydroxybiphenyl colorimetry according to a previously described method [16]. Acetic acid, propionic acid, butyric acid, and ethanol were measured using gas chromatography (Agilent 6890N, Santa Clara, CA, USA). The temperature of input was 250°C, the split ratio was 10:1, and the input volume was 1 *μ*L. Ammonia nitrogen (NH_3_-N) was quantified using the phenol-sodium hypochlorite colorimetric method [17]. The total nitrogen and crude protein contents were determined using the Kjeldahl method. Crude protein contents were calculated as 6.25 multiplied by total nitrogen. The dry matter content was determined by drying in an oven at 65°C for 48h [18]. NDF and acid detergent fibers (ADF) were measured using the Van Soest fiber wash method. The starch content was quantified using the enzymatic hydrolysis method [19]. The WSC content was determined using the anthrone-sulfuric acid colorimetric method [18]. The crude fat content was determined using the Soxhlet extraction method [20]. All measurements were repeated for three times.

### 2.5. Aerobic Stability Detection

CO_2_ gas generators were assembled as described by a previous study [21]. Each installation includes two beverage bottles of 550 mL. The upper part was added 25g silage and the lower part was added 20 mL 20% KOH. The silage samples from the opened silos were placed into the gas generators and kept at 30°C in a constant temperature incubator. Measurements of the CO_2_ production were obtained at 2, 4, 6, 8, and 10 days.

### 2.6. Comprehensive Value Evaluation

A comprehensive value evaluation was conducted using the principal component analysis model [22] to analyse the principal components of several categories of indicators, namely, the number of fungi, fermentation characteristics, aerobic stability, nutrient value, and nutrient degradation rate. The main component score was* F*_*i*_ and the principal component score was* F*. The formulas used were as follows:

(1) F=∑i=1nFiλiFi=∑i=1nAijZij

where* A *is the feature vector value and* Z* is the normalized value of each index of the corn silage, and* λ*_*i*_ represents the proportion of the variance contribution rate of the* i*th principal component to the total extraction variance contribution rate (i.e., weight), with* i *= 1, 2, 3, 4…*n*.

### 2.7. Statistical Analysis

*F*-tests were performed using SPSS 22.0 software, followed by one-way analysis of variance (ANOVA) and two-way ANOVA tests. One-way ANOVA was used to calculate the statistically significant differences between treatments, whereas two-way ANOVA was used to calculate the significance of the interactions between the time and treatments. Duncan's method was performed for multiple comparisons. All differences were considered significant at* P* < 0.05 and extremely significant at* P *< 0.01.

## 3. Results

### 3.1. *Effect* of Combined Bacterial Inoculants on Changes in the Main Microbial Flora during Silage Fermentation

Different silage fermentation groups inoculated with different microbial strains were set up to investigate the changes in the microbiota during ensiling. As presented in Table 1, the number of aerobic bacteria, LAB, yeast, and mold at 0 d was 8.19 log cfu g^−1^, 8.44 log cfu g^−1^, 6.10 log cfu g^−1^, and 5.77 log cfu g^−1^. Along with the extension of fermentation time, the number of these four bacteria fluctuated, but both showed a downward trend. The number of aerobic bacteria in YX treatment at the 10th d and 15th d was significantly higher than that in Y treatment (P < 0.05). At the 8th d and 15th d, the number of LAB in YX treatment was significantly higher than that in K treatment and Y treatment (*P* < 0.05), and, at the 45th d, it was significantly higher than that in Y treatment. The number of yeast in YX treatment at the 4th d, 10th d, 15th d, 25th d, and 35th d was significantly higher than that in Y treatment or K treatment (*P* < 0.05). The number of mold in YX treatment was significantly higher than that in K treatment and Y treatment at the 4th d, 15th d, 25th d, 35th d, and 45th d (*P* < 0.05).

Two-way ANOVA was used to calculate the significance of the interactions between the time and treatments. The interactions between treatment (M) and fermentation time (D) have extremely significant effects on the number of yeast and mold (*P* < 0.01), whereas the number of aerobic bacteria and LAB was not significantly affected by the interactions between M and D.

### 3.2. Effects of Combined Bacterial Inoculants on the Fermentation Characteristics and Aerobic Stability of Corn Silage

Both the pH value and NH_3_-N content are direct indicators of the silage condition and quality. Silage with a pH between 3.8 and 4.5 and with a low NH_3_-N content is considered of good quality [23]. As shown in Table 2, during the ensiling process, all the different types of treatments had affected the silage quality indicators significantly, including the WSC, lactic acid, and NH_3_-N contents and pH values (*P* < 0.01), but they had no significant impact on the acetic acid amounts. The fermentation time also participated in regulating the fermentation characteristics (*P* < 0.05). However, the composite fermentations with CDB and heterolactic LAB failed to affect the content of ethanol during fermentation. Besides, both propionic acid and butyric acid were not detected in the whole fermentation process.

**Figure 1:**
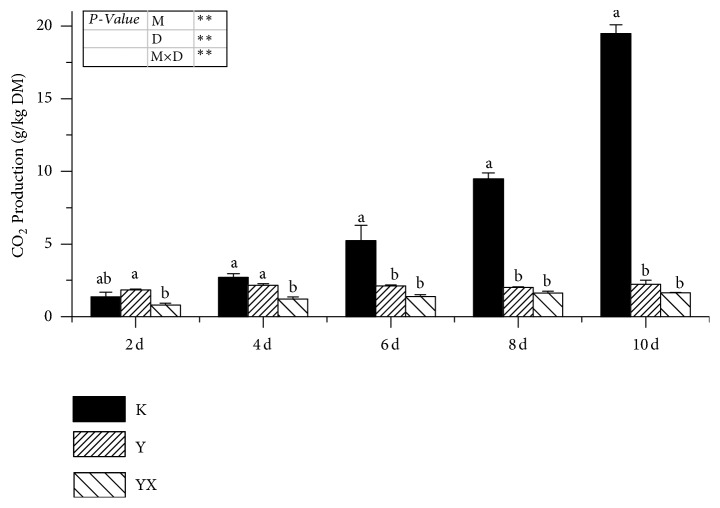
*Effects of cellulose-decomposing bacteria and heterolactic lactic acid bacteria on CO*_*2*_ *gas production in corn silages within 10 days after opening the fermentation bag.* K, untreated corn silage with no inoculant applied; Y, heterolactic lactic acid bacteria; YX, heterolactic lactic acid bacteria and cellulose-decomposing bacteria; M, treatment; D, day; M × D, treatment × day; DM, dry matter. Columns with different letters are significantly different at the 5% level using Duncan's test. *∗∗ P* < 0.05.

All the inoculant treatments and the exposure times affected the CO_2_ production markedly (Figure 1). However, there was no difference between the LAB-alone and combined CDB and LAB groups, indicating that the addition of CDB was unable to change the CO_2_ production by the original LAB inoculants.

### 3.3. Effects of Combined Bacterial Inoculants on the Nutrient Content of Corn Silage

As shown in Table 3, in general, the nutrient composition of corn silage decreases markedly at the initial stage of fermentation, but the loss of nutrients tends to progress gradually as the fermentation progresses. During the fermentation process, all the different inoculant treatments affected the nutritive index of the corn silage distinctly.

The interactions between treatment and fermentation time have extremely significant effects on the nutritive index (*P* < 0.01) except for the day matter (*P* > 0.05).

### 3.4. Comprehensive Evaluation

Principal component analysis was applied to make a comprehensive evaluation of the microbial combinations. Each treatment was ranked by value on the 60th day of fermentation, resulting in the following order: YX treatment (0.20) > control K (–0.35) > Y treatment (–0.51). Based on a comprehensive evaluation, the CDB and heterolactic LAB combination had the best effect on the ensiling process in improving the quality and feed value of corn silage.

## 4. Discussion

The major objectives of the silage and feed industry were to improve the nutritional quality, aerobic stability, and absorption efficiency of silage plants. LAB have been commonly applied as the main microbial inoculant for improving the nutritional quality of fermented silage [4]. However, with the development of the feed industry, traditional LAB-based fermentation additives are unable to meet the current industry requirements. CDB have been applied to promote the quality of fodder in recent years and have displayed good performance. In this study, the effects of combinations of CDB and heterolactic LAB inoculants on the quality, aerobic stability, and nutrient content of corn silage were studied. Through statistical analysis of various fermentation indicators, we finally concluded that the CDB and heterolactic LAB combination had the best effect on silage fermentation, promoting the quality and feed value of the fermented product.

The characterization of LAB isolated from forage crops and grasses, such as corn, alfalfa, clover, sainfoin, and timothy has been reported widely [24]. During the stable stage, both the exogenous additives and the internal bacterial species had reached an equilibrium state, thereby ensuring the long-term storage of the silage. The combined effects of the CDB and LAB had increased the number of molds in the fermentation system, thereby further increasing the number of CDB, which promoted fiber degradation [25, 26] and increased the corn silage quality.

Fermentation with the CDB and heterolactic LAB combination significantly changed the LAB counts, indicating that the addition of multiple LAB could promote the proliferation of aerobic LAB that self-convert to facultative anaerobes under aerobic conditions. The addition of CDB resulted in the rapid consumption of oxygen at the beginning of silage fermentation, which accelerated the progression of the process into an anaerobic environment. The oxygen reduction further created favorable conditions for the rapid propagation of LAB, thereby inhibiting the growth of other spoilage bacteria. On the bases of our results and previous conclusion, we speculated that controlling the time of CDB action after silo opening had a positive effect on the feeding value of the livestock that consumed the silage product.

During the fermentation stage, the YX treatment group had a significantly higher pH value and NH_3_-N contents than those of the Y treatment group. These data indicated that the combined effect of the CDB and heterolactic LAB had reduced the substrate consumption by the fermenting microorganisms and increased the acid-producing ability of the LAB.

Compared with the K treatment and Y treatment, the YX treatment increased the NDF content, ADF content and EE, indicating that the different CDB strains had different abilities in degrading the cellulose material in the silage [27, 28]. Therefore, the fermentation process should be optimized in order to enhance the enzyme production abilities of the diverse bacterial strains.

## 5. Conclusions

The regularity of the composite fermentation was revealed, and the optimal combination of bacteria was evaluated. Based on a comprehensive evaluation, the CDB and heterolactic LAB combination had the best effect on the ensiling process in improving the quality and feed value of corn silage. However, more research is still required for a comprehensive evaluation of the silage system. Notwithstanding these future investigations, our present results provide important insights that can be applied to the future development of microbial inoculants for the fermentation of livestock feed of high quality.

## Figures and Tables

**Table 1:** The change rule of cellulose-decomposing bacteria and lactic acid bacteria on the main microbial community of corn silage within 60 days (log cfu/g FM).

Day	Treatment	Aerobic bacteria	Lactic acid bacteria	Yeast	Mold
0d		8.19	8.44	6.10	5.77
2d	K	8.86	8.93	3.84	4.95
	Y	8.72	8.86	4.08	4.37
	YX	8.76	8.99	3.72	4.53
	SEM	0.06	0.05	0.31	0.14
4d	K	8.62	8.68	3.66^ab^	< 2.00^b^
	Y	8.60	8.78	3.35^b^	2.49^b^
	YX	8.73	8.71	3.70^a^	4.30^a^
	SEM	0.08	0.04	0.06	0.17
6d	K	8.66	8.73	3.48	< 2.00
	Y	8.55	8.69	4.35	< 2.00
	YX	8.77	8.74	3.59	< 2.00
	SEM	0.07	0.06	0.43	- -
8d	K	8.14	8.26^b^	3.11	< 2.00
	Y	8.30	8.30^b^	3.32	< 2.00
	YX	8.49	8.82^a^	3.38	< 2.00
	SEM	0.10	0.07	0.10	- -
10d	K	8.29^ab^	8.34	3.16^a^	< 2.00
	Y	7.77^b^	8.24	2.60^b^	< 2.00
	YX	8.77^a^	8.59	3.63^a^	< 2.00
	SEM	0.10	0.10	0.10	- -
15d	K	7.84^b^	7.68^b^	2.32^b^	3.40^b^
	Y	7.69^b^	7.57^b^	2.17^c^	4.00^b^
	YX	8.36^a^	8.31^a^	2.69^a^	4.90^a^
	SEM	0.09	0.09	0.01	0.12
25d	K	7.12	7.75	2.46^b^	3.64^c^
	Y	7.48	7.99	2.78^a^	4.05^b^
	YX	7.54	8.12	2.99^a^	4.39^a^
	SEM	0.25	0.07	0.06	0.05
35d	K	7.92	7.73	2.24^c^	3.40^b^
	Y	8.15	7.46	3.50^b^	3.70^b^
	YX	8.43	7.71	4.50^a^	4.50^a^
	SEM	0.31	0.11	0.07	0.07
45d	K	7.67	7.78^ab^	3.15	3.39^b^
	Y	7.02	7.07^b^	2.77	3.60^b^
	YX	8.07	7.97^a^	2.71	4.45^a^
	SEM	0.23	0.15	0.11	0.10
60d	K	7.40	6.89	2.46	3.16
	Y	6.58	7.37	2.48	3.31
	YX	7.42	6.99	3.23	3.86
	SEM	0.22	0.20	0.21	0.13
*P*-value	M	*∗∗*	*∗∗*	*∗*	*∗∗*
	D	*∗∗*	*∗∗*	*∗∗*	*∗∗*
	M×D	NS	NS	*∗*	*∗∗*

Note: FM, fresh matter; K, untreated corn silage with no inoculant applied; Y, heterolactic lactic acid bacteria; YX, heterolactic lactic acid bacteria and cellulose-decomposing bacteria; M, treatment; D, day; M×D, treatment × day; SEM, standard error of mean. Values followed by the different letters are significantly different at 5% level using Duncan test; *∗*, *P* < 0.05, *∗∗*, *P* < 0.01, NS, not significant.

**Table 2:** The change rule of cellulose-decomposing bacteria and heterolactic lactic acid bacteria on the fermentation characteristics of corn silage.

Day	Treatment	pH	WSC %DM	LA %DM	AA %DM	LA: AA	Ethanol %DM	NH_3_-N %DM	NH_3_-N:TN
0d		5.33	22.01	0.61	0.20	3.05	0.12	0.019	0.016
2d	K	4.47	20.00^a^	3.84^b^	3.00	1.28	0.17	0.030	0.026
	Y	4.47	18.02^ab^	4.65^a^	2.18	2.13	0.16	0.040	0.035
	YX	4.62	14.25^b^	4.10^b^	2.94	1.39	0.15	0.032	0.027
	SEM	0.03	0.71	0.06	0.39	-	0.005	0.003	-
4d	K	4.26^b^	14.02	4.72^b^	3.00^ab^	1.57	0.17	0.039	0.035
	Y	4.31^b^	14.01	7.29^a^	3.22^a^	2.26	0.16	0.028	0.025
	YX	4.56^a^	12.39	4.76^b^	2.49^b^	1.91	0.16	0.041	0.036
	SEM	0.02	0.70	0.17	0.10	-	0.003	0.003	-
6d	K	4.22^c^	12.50^a^	4.23^b^	4.66	0.91	0.17	0.049^b^	0.043
	Y	4.30^b^	4.41^b^	4.88^a^	3.47	1.41	0.17	0.054^b^	0.047
	YX	4.52^a^	6.04^b^	4.34^b^	3.28	1.32	0.17	0.077^a^	0.067
	SEM	0.01	0.29	0.10	0.36	-	0.001	0.004	-
8d	K	3.79^c^	10.99^a^	6.00	3.36	1.79	0.19	0.042^b^	0.038
	Y	3.92^b^	3.87^b^	5.59	1.52	3.68	0.16	0.058^ab^	0.053
	YX	4.28^a^	3.04^b^	5.16	1.93	2.67	0.16	0.069^a^	0.061
	SEM	0.02	0.26	0.35	0.32	-	0.011	0.003	-
10d	K	4.13^c^	11.82^a^	3.51	1.81^b^	1.94	0.17	0.084^a^	0.075
	Y	4.17^b^	3.04^b^	3.63	1.93^b^	1.88	0.16	0.061^b^	0.055
	YX	4.37^a^	3.59^b^	4.61	4.11^a^	1.12	0.16	0.076^a^	0.068
	SEM	0.004	0.32	0.33	0.35	-	0.002	0.003	-
15d	K	4.12^b^	11.13^a^	2.43^c^	3.22	0.75	0.16	0.060^b^	0.054
	Y	4.08^b^	7.39^b^	2.78^b^	2.73	1.02	0.16	0.076^b^	0.067
	YX	4.35^a^	2.54^c^	3.44^a^	2.60	1.32	0.16	0.140^a^	0.121
	SEM	0.01	0.49	0.06	0.17	-	0.002	0.007	-
25d	K	4.08^b^	6.51^ab^	2.41^b^	2.40^ab^	1.00	0.15	0.057^b^	0.052
	Y	4.12^b^	8.80^a^	2.62^b^	2.66^b^	0.98	0.16	0.067^b^	0.060
	YX	4.35^a^	5.55^b^	3.57^a^	2.97^a^	1.20	0.15	0.098^a^	0.087
	SEM	0.01	0.49	0.09	0.22	-	0.004	0.003	-
35d	K	4.02^c^	5.34	1.42^c^	1.29	1.10	0.15	0.044^b^	0.040
	Y	4.09^b^	6.64	1.77^b^	2.41	0.73	0.14	0.057^b^	0.051
	YX	4.26^a^	5.93	3.07^a^	1.41	2.18	0.14	0.121^a^	0.109
	SEM	0.004	0.25	0.05	0.28	-	0.006	0.004	-
45d	K	4.29^a^	8.44^a^	2.70	1.45	1.86	0.15	0.128^ab^	0.115
	Y	4.12^b^	5.69^b^	2.57	2.39	1.08	0.14	0.107^b^	0.096
	YX	4.25^a^	5.47^b^	3.59	1.05	3.42	0.16	0.157^a^	0.138
	SEM	0.02	0.33	0.19	0.42	-	0.006	0.005	-
60d	K	4.09^b^	5.49^a^	1.97^b^	2.18^ab^	0.90	0.14	0.075	0.066
	Y	3.98^c^	3.34^b^	2.26^b^	1.39^b^	1.63	0.21	0.119	0.107
	YX	4.27^a^	3.92^b^	3.54^a^	2.89^a^	1.22	0.63	0.126	0.112
	SEM	0.02	0.17	0.11	0.25	-	0.092	0.009	-
*P*-value	M	*∗∗*	*∗∗*	*∗∗*	NS	-	NS	*∗∗*	-
	D	*∗∗*	*∗∗*	*∗∗*	*∗∗*	-	*∗∗*	*∗∗*	-
	M×D	*∗∗*	*∗∗*	*∗∗*	*∗*	-	*∗∗*	*∗∗*	-

Note: DM, dry matter; WSC, water soluble sugar; LA, lactic acid; AA, acetic acid; TN, total nitrogen; K, untreated corn silage with no inoculant applied; Y, heterolactic lactic acid bacteria; YX, heterolactic lactic acid bacteria and cellulose-decomposing bacteria; M, treatment; D, day; M×D, treatment × day; SEM, standard error of mean. Values followed by the different letters are significantly different at 5% level using Duncan test; *∗*, *P* < 0.05, *∗∗*, *P* < 0.01, NS, not significant.

**Table 3:** The change rule of cellulose-decomposing bacteria and heterolactic lactic acid bacteria on the nutriment of corn silage (% DM).

Day	Treatment	DM	Starch	CP	NDF	ADF	EE	Ash
0d		42.27	27.73	7.31	50.09	29.14	6.68	10.69
2d	K	35.35	26.89^a^	7.21	41.42^b^	23.20^b^	7.91	8.67^b^
	Y	35.69	21.95^b^	7.15	47.93^a^	26.51^a^	7.54	12.42^a^
	YX	34.47	26.28^ab^	7.24	48.92^a^	27.46^a^	7.83	10.90^a^
	SEM	0.34	0.78	0.03	0.42	0.44	0.16	0.31
4d	K	33.56	21.00	6.98^ab^	43.85	26.85^b^	10.33^b^	8.92^b^
	Y	35.01	24.69	6.95^b^	43.48	29.45^a^	13.18^a^	13.00^a^
	YX	33.70	25.54	7.21^a^	43.26	30.66^a^	9.06^c^	9.97^b^
	SEM	0.38	1.37	0.04	0.76	0.22	0.11	0.40
6d	K	33.35	16.87^a^	7.18	39.45^c^	27.33	9.08^a^	9.56
	Y	34.78	14.02^b^	7.21	46.86^b^	29.32	6.60^b^	11.35
	YX	34.96	14.02^b^	7.19	50.76^a^	29.30	8.87^a^	8.38
	SEM	0.28	0.20	0.03	0.58	0.51	0.24	0.62
8d	K	35.58	19.20^a^	6.92^ab^	43.65	29.08^b^	9.41^b^	9.26
	Y	35.59	17.96^a^	6.87^b^	41.67	31.91^a^	10.71^a^	10.48
	YX	35.78	13.00^b^	7.06^a^	43.70	33.24^a^	10.21^a^	10.54
	SEM	0.40	0.77	0.02	0.63	0.43	0.09	0.22
10d	K	36.41	21.03	7.05^a^	46.68^a^	20.61^b^	9.18^c^	10.11^b^
	Y	36.69	21.75	6.94^b^	38.58^b^	23.32^b^	10.82^b^	12.23^a^
	YX	36.08	19.72	6.96^ab^	44.81^a^	30.11^a^	13.18^a^	10.18^b^
	SEM	0.47	0.42	0.02	0.57	0.96	0.18	0.24
15d	K	33.95^b^	15.92^a^	7.02^b^	43.34^b^	24.11^b^	6.33^a^	10.73^b^
	Y	36.25^a^	14.76^a^	7.10^ab^	38.23^c^	23.58^b^	5.50^b^	10.47^b^
	YX	34.94^b^	12.28^b^	7.20^a^	47.36^a^	27.05^a^	5.01^c^	11.84^a^
	SEM	0.23	0.32	0.02	0.47	0.16	0.07	0.14
25d	K	34.59^ab^	16.85^b^	6.93	41.66^b^	27.83^b^	6.83^b^	10.82
	Y	36.01^a^	19.59^a^	7.05	38.07^c^	25.14^b^	6.20^c^	11.53
	YX	34.08^b^	14.26^c^	7.06	46.05^a^	34.31^a^	7.45^a^	10.67
	SEM	0.30	0.26	0.02	0.49	0.58	0.08	0.26
35d	K	34.70	13.78^b^	6.79^b^	43.81	25.65	4.70	9.48^b^
	Y	33.82	12.35^c^	7.05^a^	45.12	26.93	4.73	11.67^a^
	YX	32.84	15.57^a^	6.97^a^	43.07	28.31	4.83	10.64^ab^
	SEM	0.45	0.16	0.02	0.69	0.89	0.06	0.27
45d	K	34.69^a^	18.13^a^	6.91	43.77^b^	28.25	6.97	10.25
	Y	33.86^ab^	12.85^b^	6.98	48.17^a^	30.08	6.25	10.61
	YX	32.24^b^	13.95^b^	7.07	42.30^c^	31.75	6.48	11.63
	SEM	0.32	0.31	0.03	0.19	0.70	0.16	0.23
60d	K	33.86^b^	15.78	7.05	45.87^a^	27.53	8.01^b^	8.33^b^
	Y	35.89^a^	16.10	6.93	42.85^b^	25.29	8.14^b^	10.70^a^
	YX	34.62^ab^	15.36	7.05	41.47^c^	27.10	10.25^a^	10.83^a^
	SEM	0.28	0.40	0.02	0.22	0.57	0.09	0.31
*P*-value	M	*∗∗*	*∗∗*	*∗∗*	*∗∗*	*∗∗*	*∗∗*	*∗∗*
	D	*∗∗*	*∗∗*	*∗∗*	*∗∗*	*∗∗*	*∗∗*	NS
	M×D	NS	*∗∗*	*∗∗*	*∗∗*	*∗∗*	*∗∗*	*∗∗*

Note: DM, dry matter; CP, crude protein; NDF, neutral detergent fiber; ADF, acid detergent fiber; EE, ether extract; K, untreated corn silage with no inoculant applied; Y, heterolactic lactic acid bacteria; YX, heterolactic lactic acid bacteria and cellulose-decomposing bacteria; M, treatment; D, day; M×D, treatment × day; SEM, standard error of mean. Values followed by the different letters are significantly different at 5% level using Duncan test; *∗P* < 0.05, *∗∗P* < 0.01, NS, not significant.

## Data Availability

It is not applicable. This study was only the primary research, and further study has been in progress.
